# Preclinical Models of Nontuberculous Mycobacteria Infection for Early Drug Discovery and Vaccine Research

**DOI:** 10.3390/pathogens9080641

**Published:** 2020-08-06

**Authors:** Elisa Rampacci, Valentina Stefanetti, Fabrizio Passamonti, Marcela Henao-Tamayo

**Affiliations:** 1 Department of Veterinary Medicine, University of Perugia, Via San Costanzo 4, 06126 Perugia, Italy; elisa.rampacci@gmail.com (E.R.); valentina.stefanetti@unipg.it (V.S.); 2 Mycobacteria Research Laboratories, Department of Microbiology, Immunology, and Pathology, Colorado State University, Fort Collins, CO 80523, USA; Marcela.Henao_Tamayo@colostate.edu

**Keywords:** nontuberculous mycobacteria, drug susceptibility testing, vaccine, biofilm, flow-cytometry, hollow-fiber system, in vitro and in vivo models

## Abstract

Nontuberculous mycobacteria (NTM) represent an increasingly prevalent etiology of soft tissue infections in animals and humans. NTM are widely distributed in the environment and while, for the most part, they behave as saprophytic organisms, in certain situations, they can be pathogenic, so much so that the incidence of NTM infections has surpassed that of *Mycobacterium tuberculosis* in developed countries. As a result, a growing body of the literature has focused attention on the critical role that drug susceptibility tests and infection models play in the design of appropriate therapeutic strategies against NTM diseases. This paper is an overview of the in vitro and in vivo models of NTM infection employed in the preclinical phase for early drug discovery and vaccine development. It summarizes alternative methods, not fully explored, for the characterization of anti-mycobacterial compounds.

## 1. Introduction

Bacteria different from *Mycobacterium tuberculosis* but equally belonging to the *Corynebacteriales* order can produce chronic cavitary disease in humans and animals, a clinical characteristic of pulmonary tuberculosis (TB). From the *Corynebacteriales* group, pulmonary infections caused by *Rhodococcus equi* and nontuberculous mycobacteria (NTM) are increasingly being detected as causatives of cavitary disease [1]. In particular, NTM are responsible for severe animal and human diseases, which are notoriously challenging to treat. NTM are widely distributed in the environment and while, for the most part, they behave as saprophytic organisms, in certain situations, they can be pathogenic, so much that the incidence of NTM infections has surpassed that of TB in developed countries [2]. The collective name NTM is given to a variety of microorganisms, among which slow-growing *Mycobacterium avium complex* (MAC), *Mycobacterium ulcerans*, *Mycobacterium xenopi*, *Mycobacterium kansasii*, fast-growing *Mycobacteroides abscessus*, and *Mycolicibacterium fortuitum* stand out for increasing incidence and dangerousness [3]. NTM are comprised of tens of bacterial species. Table 1 lists some of the most common NTM and their host-associated tissue tropism. While chronic pulmonary disease is the most common localized illness caused by NTM in humans, extrapulmonary conditions include lymphadenitis and soft tissue infections in both animals and humans. Paratuberculosis, caused by the NTM *Mycobacterium avium* subsp. *paratuberculosis* (MAP) is a significant illness for its impacts on the economy, animal welfare, and public health. Dermatologic diseases caused by NTM are increasingly recognized both in human and veterinary medicine [4,5]. The incidence among humans is on the rise as the consequence of a growing immunocompromised population and people with pre-existing lung conditions; women and older individuals have a higher risk as well [6,7]. The same is true in companion animals undergoing immunosuppressive therapy [8,9,10]. The importance of NTM infections as a differential diagnosis, even in immunocompetent individuals and animals, is highlighted in the literature [11,12]. Other risk factors include the close relationship of owners and pets as a possible environmental reservoir for mycobacteria [11]. Over time, a growing body of the literature has focused on the crucial role that drug susceptibility tests (DSTs) play in the design of appropriate therapeutic regimens against NTM diseases. Currently, no standardized antimicrobial treatment exists due to the diversity of NTM infections, which calls for different approaches. This review describes the in vitro and in vivo models of NTM infection proposed in the preclinical phase for early drug discovery and vaccine development, also including mycobacterial biofilms as a target. In addition to the traditional guidelines for NTM DST, rapid flow-cytometry-based methods and pharmacokinetic/pharmacodynamic (PK/PD) in vitro simulations are presented as well. Records were identified through PubMed database searching and Clinical and Laboratory Standards Institute (CLSI) documents according to Supplementary Figure S1.

## 2. Extracellular Susceptibility Testing: The High-Throughput Way for Early Drug Discovery

Measuring the capability to inhibit the bacterial growth is the most straightforward way to test drug efficacy. By definition, such an experimental approach can exclusively address replicating bacteria. Such a principle is the basis of extracellular high throughput screens and assays that, as a result of their standardization, should be done on clinically significant isolates of certain NTM. This methodology has to serve as a comparative model when novel methods to evaluate the activity of drugs are introduced. Drug susceptibility methods are traditionally different for rapidly growing mycobacteria (RGM) vs. slowly growing nontuberculous mycobacteria (SGM). Thorough methodological guidelines can be found in the CLSI document M24, whereas M62 includes interpretative breakpoints and quality controls [13,14]. The CLSI recommends the standard broth microdilution method to determine the susceptibility of SGM and RGM to antimicrobials based on the definition of the minimum inhibitory concentration (MIC). Table 2 shows information about the culture conditions and length of the assays. Varying the growth condition is required in some instances, such as for *M. xenopi* and *M. malmoense* that may need to be incubated for three to four weeks or at a higher temperature (42 °C). Moreover, incubation for up to 14 days may be needed to reliably detect clarithromycin and amikacin resistance in *M. abscessus* complex isolates.

The CLSI recommends to cautiously interpret the MIC provided by the broth microdilution procedure for RGM. A positive result is associated with moderate countable colonies with slight turbidity, definite turbidity and clumpy growth, and definite haze or turbidity. Conversely, a few countable colonies with or without hazy turbidity provide uncertain results. In the event of uncertainty, care must be taken to read known negative wells that may appear as a slight precipitate related to the inoculum.

The CLSI suggests performing the broth microdilution manually. Nevertheless, there is currently a commercial pre-dosed, ready-to-use microdilution technique, known as RAPMYCO, designed specifically for RGM and aerobic actinomycetes. Alternatively, the MICs can be measured by performing the E-test. This method is based on the use of a predefined gradient of antimicrobial concentrations on a strip applied onto the agar surface. However, neither E-test nor an agar disk elution and agar disk diffusion has been standardized for RGM or SGM [15], and thus, they should be used only as an adjunct to broth microdilution test.

Drug susceptibility testing for SGM is notoriously challenging. The complicated inoculum calibration, the length of the assays, and the remarkable interspecies differences and intraspecies variability are responsible for the frequently inconsistent results of susceptibility testing. The MICs against SGM have been predominantly determined by the proportions in agar, radiometric macrodilution BACTEC 460, the fluorescence-based MGIT 960 system, and the traditional broth microdilution method, including the commercial pre-dosed test SLOMYCO Sensititre. The latter method simplifies testing MICs against clinical SGM. However, some limitations have been pointed out, particularly poor reproducibility [16]. The radiometric BACTEC 460 instrumentation has been extensively used in the past for susceptibility testing of SGM. Nowadays, in clinical laboratories, the fully automated nonradiometric system BACTEC MGIT 960 has mostly replaced the radiometric method for the isolation and susceptibility testing of pathogenic mycobacteria [17]. Additionally, MGIT960 may be used for the early detection of active NTM growth based on the rapid analysis of mycolic acids by high-performance liquid chromatography after 72 h of incubation [18]. On the other hand, automatic methods can be prohibitively expensive for underdeveloped countries. Therefore, isothermal microcalorimetry has been proposed as an easy and relatively inexpensive alternative for MIC determination [19]. Rapid colorimetric DSTs based on the use of Alamar blue, water-soluble tetrazolium salts, and resazurin have been exploited to determine MICs at a reasonable cost [20,21,22,23]. By determining metabolic activity, these approaches may be used to assay compound efficacy against non-replicating mycobacteria, which are notoriously difficult to treat [24]. Table 2 lists some of the most frequently used high throughput extracellular assays for NTM DST.

Finally, by performing time-kill assays, researchers can obtain some pharmacodynamic information for critical antibiotics. Such experiments could require long drug exposure time, depending on the mycobacterial species tested. Therefore, a preliminary investigation on drug stability in culture medium is recommended.

The aforementioned rapid screens are considered the first step for building an optimized chemotherapy regimen. However, the use of the single extracellular assay is not sufficient to select an effective therapeutic program, due to the complexity of NTM disease and the intracellular pathogenesis.

## 3. Static Intracellular Infection Models

### 3.1. Immortalized and Primary Cells for In Vitro Intracellular Assays

It is common knowledge that NTM are successful facultative intracellular pathogens, capable of surviving and persisting within the host macrophages [25]. This fact suggests that the search for anti-NTM antibiotics should include methods to identify those effective intracellularly. As highlighted in Table 3, intracellular DSTs can differ in selected cell types and experimental procedures. The human monocytic cell lines THP-1 and U937 are commonly used to screen antimicrobial molecules and investigate dose-response against intracellular NTM [26,27,28,29,30]. These cells are differentiated by phorbol 12-myristate 13-acetate (PMA) to mimic the morphology and functions of mature macrophages. When required, THP-1 and U937 can be activated by treatment with interferon-γ (IFNγ) to undergo proinflammatory response [31]. This methodological approach is widely applied to avoid sampling of human specimens and increase assay reproducibility. However, unveiled discrepancies exist between cell lines and primary human monocyte-derived macrophages (hMDM). Indeed, the phagocytosis activity of mycobacterial cells exerted by resting and IFN*γ*-activated U937 macrophages was significantly lower than that observed in hMDM and THP-1 macrophages [32]. Additionally, PMA-induced maturation of U937 and THP-1 cell lines alters the surface markers, the transcriptomic profile, and the cytokine production pattern, inducing a significant TNFα production in resting macrophages [33,34]. This notwithstanding, whereas U937 macrophages appeared to differ substantially from hMDMs, THP-1 cells are assumed to be representative of the macrophages. THP-1 and hMDMs showed similar bacterial phagocytosis and host response to infection with *M. tuberculosis*, as well as similar cytokine/chemokine type and secretion [35]. Despite the lack of comparative studies that specifically investigate NTM-host response in different cellular substrates, the majority of researches have employed THP-1 cells for DST on NTM. Otherwise, the human A549 alveolar cell line has been used to a limited extent as a specific model of pulmonary epithelial infection [36,37,38].

Murine macrophagic cell lines, such as RAW264.7 and J774, should be preferred to select molecules for a subsequent in vivo study in murine models [39,40,41,42,43]. Regardless of the type of cell line selected, the inclusion of the cytotoxicity test for the antimicrobial of interest against the same cell line is indispensable for interpreting the results. Most commonly, shorter assays, consisting of 24–48 h of drug exposure, are preferred to ensure the survival of macrophages until the end of the test. However, cytotoxicity assays revealed consistent viability up to day 16 post-infection of THP-1 cells with mycobacterial cells [35]. In the event of an extended length of the assay, the estimation of cell viability and analysis of drug stability are recommended.

More recently, primary cell assays have been used only in confirmatory studies [35]. In the past, there were numerous researches to investigate drug efficacy in human peripheral blood mononuclear cells (PBMCs) [30,44,45,46,47,48]. For studies involving mice, primary bone marrow-derived macrophages have been preferred to other primary cell cultures [49,50], although some older studies have also suggested the use of zymosan A-induced murine peritoneal macrophages [51,52].

Finally, another promising line of research would be establishing the suitability of the Max Planck Institute (MPI) cells for in vitro studies of respiratory disease caused by NTM. MPI cells are murine primary cells than can be propagated continuously [53]. Being functionally very close to lung alveolar macrophages, MPI cells have been proposed for understanding the functions of alveolar macrophages in TB and to determine the anti-TB drug activity [54].

### 3.2. Models Resembling In Vivo Pathological Conditions

As is known, immunosuppression is a contributing factor for mycobacterial infections [7]. As a result, some authors have introduced useful in vitro intracellular models of mycobacteria and HIV-1 co-infection [55,56]. This strategy could be used to preliminary screen compounds from large chemical libraries, before proceeding to evaluate safety and efficacy in challenging animal models. A further advantage of in vitro intracellular models consists of the possibility to investigate potential states promoting susceptibility to mycobacterial infections in the host, such as diabetic glucose levels [57] or low PO_2_ [58]. Historically, mycobacterial infections are related to granuloma formation. As a result, the importance of developing models resembling granulomatous infections has been discussed in the literature [59,60]. The proposed models showed similar cellular differentiation to natural granulomas and recapitulated molecular mechanisms established in corresponding diseased tissues. These findings suggest opportunities for future research specifically addressed on NTM-induced granulomas, as done for MAP [61].

### 3.3. Methods to Estimate Mycobacterial Killing

Colony-forming unit (CFU) enumeration has remained the most widely used method for determining growth on selective agar media after drug exposure and cell disruption by sodium dodecyl sulfate or deionized water. However, the CFU-based method is time-consuming and could provide inconsistent results. Therefore, luminescence-based strategies have been proposed for rapid intracellular assays, particularly for testing antitubercular drugs [62,63,64]. Additionally, several methods for DST by flow cytometry have been developed, and more details are given below. Even if a closer look at the literature on luminescence-based methods for NTM DST reveals some gaps, previous research [65] has the potential to promote future studies on this topic.

To sum up, in this section, we reviewed the strategies of DST exploiting mycobacteria-infected static cellular models. Even though a number of weak points need to be considered, the results achieved through these methods are substantial and paved the way for significant progress in anti-mycobacterial drug discovery and development.

Examples include, but are not limited to, novel drug delivery systems, as is the case of nebulized liposomes for pulmonary targeting [26,42,43,48,66,67,68,69], nano-formulations and polymeric particles [64,70,71], and chemical compounds restoring antimicrobial susceptibility, among which efflux pump inhibitors deserve to be mentioned [72,73].

## 4. Flow-Cytometry-Based Drug Susceptibility Testing

Rapid flow cytometry-based assays have been proposed for determining the antimicrobial susceptibility of mycobacteria within a few hours of antibiotic exposure. The majority of flow cytometry-based DSTs were performed against *M. tuberculosis*, while these have been previously tested only to a limited extent against NTM. The first flow cytometry methods based on the principle that treated tuberculous and nontuberculous mycobacteria hydrolyze significantly less fluorescein diacetate to free fluorescein than non-treated bacteria, which therefore demonstrate less intensity of fluorescence [75,76,77,78]. The strength of these assays is to provide results within 24 h, thanks to the fact that they did not require multiplication of the mycobacteria. On the other hand, safety was a significant concern, because mycobacteria were not killed before the flow cytometric analysis. Therefore, flow cytometric susceptibility was performed by biologically safe enumeration of unstained *M. tuberculosis* and *M. avium* inactivated by paraformaldehyde [79,80,81]. However, unstained particles may result in low accuracy. As a result, the use of fluorescent nucleic acid stains was proposed, using either the single green fluorescent staining SYTO [82,83] or multiple dyes. This latter approach can be used to distinguish live, injured, and dead cells by using SYTO 9/Propidium iodine for live/dead discrimination and ethidium monoazide, plus visible light irradiation. Such a treatment cleaves DNA exclusively of antibiotic-injured bacteria, and the resultant decrease in the spaces of DNA base pairs inhibits the intercalation of SYTO9 at least of an 80% [84]. Secondly, multiple-fluorescence flow-cytometry can differentiate between mycobacterial populations growing at different rates and investigate the mode of action of different antibiotic classes. Indeed, antibiotics targeting the cell wall show distinct fluorescence profiles from those inhibiting intracellular processes. In this regard, one method exploited calcein violet with an acetoxy-methyl ester group and Sytox green that distinguish live cells and damaged bacteria, respectively, to obtain useful differential patterns of fluorescence and characterize subpopulations of antibiotic-treated microorganisms [85]. The tendency of mycobacteria to clump could create problems with flow cytometry analysis. To overcome the problem, encapsulating the bacilli in gel micro-drops before analysis was proposed as an alternative to traditional techniques for rapid susceptibility testing of SGM [86,87]. Later, some authors drove the development of modern flow cytometry-based DST employing fluoromycobacteriophages—viruses of mycobacterial hosts containing fluorescent genes, such as *gfp* or *ZsYellow* [88,89]. The addition of selected antibiotics simultaneously with phage to a susceptible bacterium obliterates fluorescence, whereas the fluorescence profile of a resistant strain is not altered from untreated cells by the addition of the phage. However, specific studies have almost exclusively focused on *M. tuberculosis* [90].

In short, reviewing the literature about the application of flow cytometry-based DSTs has pointed out the advantages to

1. Obtaining sensitive and reproducible results within a few hours of drug exposure;
2. Testing the susceptibility of the whole population, including viable but not culturable mycobacteria. These microorganisms cannot be cultivated on agar. As a result, the conventional methods can underestimate the enumeration of cells within a bacterial population;
3. Discerning the way novel antibiotics work by distinctive patterns of fluorescence;
4. Screening for new drugs against intracellular pathogens using imaging flow cytometry in combination with dedicated software. This methodology represents a powerful new approach to investigate host cell-pathogen interaction, including bacterial internalization and localization [91].

Nevertheless, the required equipment might be prohibitively expensive for routine use in developing countries where mycobacterial diseases are a health challenge.

## 5. Dynamic Cellular Infection Models

PK/PD properties of molecules under development have been assessed only to a limited extent, because of the high cost of suitable animal models and the practical and financial difficulties of performing PK studies in patients. To optimize dosing regimens with pharmaceutical candidates early in the development process, most initial studies and current works focus on developing tools for PK/PD in vitro simulation. A close look at the literature reveals the two-compartment hollow-fiber bioreactor system (HFS) as the most used PK/PD modeling of NTM pulmonary infection. The method is based on the use of a hollow fiber cartridge containing thousands of small tubular fibers, through which the medium is pumped. The first hollow-fiber system model of intracellular pulmonary MAC (HFS-MAC) was developed in 2010 by Deshpande and colleagues [92]. Experiments with HFS-NTM were planned to identify

1. The inhibitory sigmoid maximum kill (E_max_) in relation to the known pharmacokinetics achieved in the lungs;
2. The exposure associated with suppression of resistance emergence;
3. The optimal bacterial kill based on PK/PD index obtained by performing dose-fractionation studies [93].

The system dynamically mimics the human concentration-time profiles of antibiotics in the central compartment, where 10% FBS supplemented-RPMI medium circulates without cells. After inoculation into the external compartment, infected non-activated THP-1 macrophages are ceaselessly immersed in the liquid medium that freely crosses the semipermeable hollow fibers, while the cells are too big to pass across. The confinement of the macrophages to the peripheral compartment allows the collection of the cells for quantitative analysis. The HFS-MAC model was then adapted to perform PK/PD studies of drug candidates against *M. abscessus* [94] and SGM other than MAC [95]. Table 4 summarizes the applications of HFS models for NTM drug discovery. As shown, HFS proved to be useful to compare different therapeutic regimens, study pharmaceutical candidates, and drug combination synergy up to 28 days of treatment. However, the combinatorial approach is challenging to address in HFS, due to the intractable equipment required for screening the exposure-response associated with several pharmaceutical interactions. Therefore, as a preliminary step, synergy studies are conducted on multi-wells matrixes containing infected adherent macrophages [96]. After that, HFS for combination therapy is used to identify a regimen that is at least four times faster than current 18-months therapy, and that tackle the emergence of resistant bacteria [93]. Bacterial density can be adjusted to simulate the average bacterial burden in the lungs. At the same time, the Wayne-Hayes model can be applied to an anaerobic HFS-NTM for studying the effect of drugs against non-replicating persistent mycobacteria, as done for *M. tuberculosis* [97]. Such an approach mimics the lung pathologic conditions induced by TB and NTM infection, where necrosis-induced hypoxia could select for non-replicating and drug-tolerant mycobacteria. HFS is also useful to strengthen the statistical power by performing repetitive PK/PD measurements, not feasible from in vivo terminal infection models. Additionally, repeating samples allows for the monitoring of drug resistance emergence and, consequently, is the best treatment approach to tackle it. On the other hand, such an apparatus requires a highly accurate experimental design and appropriate controls to run, including the standard regimen of treatment.

The European Medicines Agency (EMA) qualified the HFS models as an integrative drug development tool for TB in combination with Monte Carlo simulations [98], which is often combined with HFS-NTM as well. Monte Carlo analysis is used to perform virtual clinical trials. This simulation generates a robust population PK model, taking account of the variability among at least 10,000 in silico patients of interest [93], and correlates it to the susceptibility of the bacteria of interest to the antibiotics. The EMA committee also encourages prospective studies in order to make the method reproducible. However, the HFS model cannot substitute for preclinical animal models or clinical trials. Indeed, one negative factor of this methodological approach is the lack of all in vivo determinants, particularly the support of the host immune system on bacterial clearance. Findings based on the use of HFS-TB have been generalized to NTM therapy, including the inhalation approach. However, while the predictive accuracy of HFS model of pulmonary tuberculosis is 94% compared with clinical data [99,100], that of the HFS-MAC is yet to be determined. This notwithstanding, HFS simulation in association with Monte Carlo analysis represents a clear advance for human treatment on previously described methods.

Conversely, in the animal health field, the use of PK/PD simulation by HFS has got attention in recent years to combat antimicrobial resistance spread. Tetracyclines have started to be evaluated in the HFS model against bovine pathogens [unpublished data, abstract], while no specific PK/PD simulation has been conducted for NTM infections in companion animals.

## 6. Anti-Biofilm Drug Development

Biofilms are frequently implicated in the pathogenesis of chronic diseases. Biofilms are immobile communities of bacteria adhering to each other and biotic or abiotic surfaces. Once attached to the surface, these bacteria start to produce a matrix mainly composed of polysaccharides, lipids, and nucleic acids [113]. NTM are notorious biofilm producers, although, unlike other bacteria, they lack surface fimbriae or pili, and they do not produce the usual exopolysaccharides as part of the biofilm matrix [114]. It has been speculated that the shorter mycolic acid chains of the mycobacterial cell wall may form the hydrophobic extracellular matrix in *M. smegmatis* biofilm [115]. Recently, it has been suggested that the hydrophobicity of NTM leads them the preferential surface adherence, rather than residence in aqueous suspension [116]. The ability of NTM to produce biofilm represents a successful survival strategy for these ubiquitous microorganisms. This behavior has been linked to their pathogenicity [117,118] and their increased tolerance to antimicrobials [119,120,121,122]. Additionally, the correlation between NTM biofilm and lung disease has been recently proved both in vitro [123] and in vivo [124,125]. However, it should be underlined that the measurement of antibiotic susceptibility in vitro has not matched with therapeutic efficacy in patients with pulmonary disease. This may be due not only to NTM “colonial variation”, but also to the abovementioned hydrophobicity. Such a property creates problems in measuring MICs, as recommended by CLSI, and makes this assay a poor predictor of clinical outcome. For this reason, several systems have been developed to measure MIC of biofilm-grown NTM cells. The first anti-biofilm drug susceptibility test utilizes 96-well polystyrene flat-bottomed microtiter plates, in which NTM can produce biofilm. After the incubation, and once rinsed away the planktonic bacteria, biofilm formation with or without antibiotics is quantified by staining with crystal violet. The solution absorbance is then measured spectrophotometrically at A_570_, and finally, the antimicrobial concentration that inhibits biofilm growth is determined [117,123]. The second rapid and reproducible assay for biofilm sensitivity to antibiotics is the Calgary Biofilm Device [126]. The minimal biofilm eradication concentration (MBEC), i.e., the minimal concentration of antibiotic required to eradicate *Mycobacterium phlei* biofilm, has been evaluated by Bardouniotis et al. [127]. The MBEC is determined by a 96-peg lid-plate and a ridged trough into that a standardized inoculum is added. At indicated time points, biofilms growth is evaluated by aseptically removing the pegs from the lid and transferring to a traditional 96-well plate, in which the biofilms are antibiotically treated. The pegs are then cleaned from biofilms through sonication in the medium and plated to determine the CFUs. Clary and colleagues have recently used a fluorescence-based method for biofilm degradation assay. They tested the inhibitory biofilm activity by measuring the relative fluorescence intensity of *mCherry*-expressing on smooth and rough strains of *M. abscessus* over time compared to untreated control [128]. Another notable assay has been recently described for *M. abscessus* by Rodríguez-Sevilla et al. They evaluated the effect of antimicrobial treatment on biofilm formation through the use of polycarbonate membranes. *M. abscessus* suspension was used to inoculate polycarbonate membrane filters (0.2 µm pore) resting on agar culture medium. Biofilm was grown and then transferred to antibiotic-containing agar and incubated. When sampled, each membrane-supported biofilm was vortexed and sonicated and cultivable bacteria were quantified by drop plating. The efficacy of antimicrobial treatment on biofilm formation was calculated as the number of CFU/cm^2^ of the membrane [129]. An important number of studies demonstrated that the NTM biofilms show increased tolerance to antimicrobials, as compared to their planktonic counterparts [120]. However, the reason is still unclear. The treatment of the biofilm seems to be more successful when used in the initial formation process, compared to the already-established biofilm [119,130]. Co-infections with other bacteria in the respiratory tract are frequently diagnosed. Therefore, the implications of mixed-species biofilms on antibiotic resistance are certainly to be investigated [116]. Additionally, non-replicating NTM are notoriously capable of surviving in unfavorable micro-environmental conditions such as oxygen and nutrient deprivation for a long time. This strategy decreases or inhibits the antibiotic efficiency targeting replicating microorganisms, and contributes to the emergence of NTM persisters, which can grow as a surface-associated biofilm. Notably, the utility of a “persister assay” for drug discovery has been recently demonstrated [131]. In an attempt to understand these resistance patterns, it has been hypothesized that the permeability of anti-TB drugs is independent among the different NTM species [122]. New therapeutic strategies with candidate anti-biofilm molecules have been proposed to improve treatment efficiency [132]. Munoz-Egea et al. demonstrated that N-acetylcysteine and Tween 80 combined with antibiotics exert a synergistic effect and are effective against RGM biofilm [133]. The degradation of biofilm matrix through enzymes such as DNase appears to be an up-and-coming tool. The presence of extracellular DNA in *M. avium* subsp. *hominissuis* biofilm matrix seems to play a role in the survival to antibiotics. As a result, the degradation of this biofilm component by DNase I allows the increased penetration of antibiotics, making the biofilm matrix a target candidate to treat NTM biofilm [134]. It is also worth mentioning that the biofilm-embedded *Methylobacterium* sp., a genus of common waterborne bacteria, has been associated with a low proportion or absence of *M. avium* [135]. Recently, it has been demonstrated that *Methylobacterium* sp. could inhibit RGM biofilm formation [136]. Such a finding suggests that the use of this microorganism could be a promising anti-biofilm strategy against some species of mycobacteria.

## 7. Animal Models in Preclinical Drug Development

### 7.1. Nonmammalian Models

*Amoebas*—several nonmammalian models of infection have been developed over the years as attractive alternatives for antimicrobial drug screening in terms of speed, cost, and ethical acceptability over the murine model. There exists a narrow body of the literature on amoebas as an experimental system for anti-mycobacterial drug testing. As environmental phagocyte organisms, amoebas, particularly *Acanthamoeba* spp., can be natural hosts of NTM, which can survive within cysts for prolonged periods [137]. Intracellular *M. avium* in *Acanthamoeba castellanii* showed higher resistance to antimicrobials, such as rifabutin, than in macrophages, thus reducing the occurrence of false-positive results of drug efficacy testing [137,138]. Therefore, *Acanthamoeba* appears to be a promising genus of amoebae for drug screening purposes. *Dictyostelium discoideum* has been the most widely used amoeba model, due to its fully sequenced haploid genome, and the easy production of mutants [139]. Since the target of mycobacterial infection is the macrophagic cell, amoebae, which are also phagocytic organisms, are a reasoned choice for host-pathogen interaction studies and to test potential pharmacological strategies. In this regard, some authors described a fluorescence-based phenotypic assay for drug screening by using the fluorescent *gfp*-expressing *Mycobacterium marinum* for infecting amoebas *A. castellanii* and *D. discoideum* in a 96-well format [140,141]. Recently, a high-content fluorescence microscopy method was adapted to *M. abscessus* grown both in THP-1 and *D. discoideum* [142]. *M. abscessus* could infect and persist in *D. discoideum* for two days without clearance or replication, as similarly reported for *M. marinum* [143]. At the same time, the host continues to replicate going over its monocytic lifestyle after this time frame. Nevertheless, the two-day exposure time was enough to observe the impact of the drug on RGM within the amoeba.

The benefits provided by the amoeba-based assays in comparison to the traditional macrophage-based screening were validated by studies on antitubercular compounds. The most remarkable result to emerge is the ability of these whole-cells to provide a host response in situ and to reveal anti-infective effects. Conversely, the main criticism of this method is related to the maximal survival temperature of *D. discoideum* (27 °C), which forces to measure the bacteria growth inside amoeba at 25 °C.

*Drosophila*—*M. abscessus* is the most drug-resistant *Mycobacterium* species. The threat posed by this species has led researchers to investigate novel infection models for in vivo drug efficacy. *Drosophila melanogaster* has been demonstrated to be a tractable model host for *M. abscessus* [144]. This system was exploited to screen several anti-*M. abscessus* drugs and possible antimicrobial combinations [145]. Tests were performed in adult female *D. melanogaster* mutant w1118 flies aged 5 to 7 days that were anesthetized and injected with a green fluorescent protein (*gfp*)-expressing *M. abscessus* ATCC 19977 between the ventral and dorsal cuticles. Following infection, the flies were incubated at 29 °C on fly medium containing antibiotics. At the end of the incubation time, the bodies were homogenized and plated for CFU enumeration and fluorescent image analysis. In similar studies, experiments were performed in adult males infected with *M. marimum* to assay anti-mycobacterial drugs [146,147]. Although *M. abscessus* and *M. marinum* demonstrated to be pathogenic when injected into *D. melanogaster*, this has been previously assessed only to a minimal extent.

*Galleria mellonella*—in recent times, an in vivo model for *M. abscessus* infection and drug testing has been established in *Galleria mellonella* larvae [148]. In addition to low cost, easy handling, and ethical acceptability, the advantages of this system include the possibility to test the bacterium in physiologic temperatures (up to 37 °C). Moreover, the histopathological model resembles that in humans, due to the presence of phagocytic nodule-forming cells, although the larvae do not possess lymphocytes. This model was further enhanced by the use of a luminescent *M. abscessus* mutated strain that simplifies the assessment of infection development and drug activity. The suitability of *G. mallonella* as a model for mycobacterial diseases was investigated through infection with *M. fortuitum*, *M. marinum*, and *M. aurum* [149]. Except for *M. aurum*, which had no detrimental effect on larval survival, the larval survival decreased as the inoculum size increased. *M. marinum* required fewer bacteria to decrease larval survival than *M. fortuitum.* Similar to other nonmammalian models, the pharmacokinetics of the antibiotic molecules in larvae are unknown. As might have been expected, the drug-exposure response may fail to emulate that observed in humans. Additionally, larvae are unlikely to be of use for chronic infection models.

*Zebrafish*—a series of recent studies have indicated the zebrafish (ZF) model (*Danio rerio*) as a valid tool in the preclinical phase of drug screening. Mycobacteria-infected ZF mimics certain aspects of mycobacterial diseases, such as the onset of granuloma-like lesions and chronic infection. Additionally, the immune system of ZF includes an early innate immunity given by macrophages and neutrophils, as well as an adaptive immunity supported by circulating lymphocytes [150,151,152,153]. As a result of its small size, ZF is easy to maintain in large numbers. Therefore, it is ideal for laboratory work, reducing the number of drug candidates to test on more costly murine models. Despite the unveiled discrepancies between the susceptibility profile of *M. marinum* and that of *M. tuberculosis*, *M. marinum*-infected ZF has been widely applied for addressing the issues related to TB drug discovery. Indeed, the capability to grow within macrophages and to produce a chronic granulomatous pathology makes *M. marinum* a surrogate candidate of TB [154,155,156,157,158,159].

With the emergence of *M. abscessus* infections, the ZF model has been proposed to image and monitor the spatiotemporal progression of the infection of *M. abscessus* in live animals and to determine the drug effect [160,161,162,163]. A typical experimental model consists of ZF larvae infected by microinjection into the caudal vein and transferred into 96-well plates, where they are exposed to various concentrations of drugs in water. The antimicrobial efficacy is determined by assessing the cardiac activity as a measure of embryo survival, and the bacterial burden by CFU enumeration after embryos lysis. Adult ZF has been used to study antimicrobial treatments against NTM as well [164]. Interestingly, genetic mutation, gamma irradiation, or immunosuppressive molecules can obliterate CD4+ T cells in adult zebrafish, mimicking the common HIV-mycobacteria co-infection [165].

The ZF offers the possibility to image host-pathogen interactions at a cellular level, due to its transparency and existing recombinant bacterial strains that express fluorescent proteins [161] or are bioluminescently tagged [157]. Additionally, bacterial burdens can be analyzed with a fluorescent pixel count by fluorescence microscopy [64]. This technique correlates well with the results of CFU count after the plating of infected embryos [166]. By combining bacteria expressing fluorescent proteins and nanoparticles containing fluorescent dye, such as coumarin [167,168], some authors were able to localize nanoparticles inside the cells and visualize fluorescent phagocytes in real-time.

This model is not intended to replace mammalian infection models. Instead, it is an attractive tool for phenotypic screenings of antimicrobial compounds and spatiotemporal observation of the pathophysiological events taking place.

### 7.2. Mammalian Models

Seminal reviews have been previously done on mammalian models of NTM infections, including *M. abscessus*, MAC, *M. kansasii*, and *M. ulcerans* [169,170,171,172,173,174]. The mouse infection model has been more extensively studied than guinea pig and rabbit in the preclinical phase of NTM drug discovery and vaccine research, as a result of transgenic and knockout line production. Most of the immunocompetent mice are not adequate animal models for drug discovery against RGM, due to transient infection with a rapid clearance after infection [161,175,176]. Therefore, the majority of prior research has exploited multiple deficits in innate and acquired immunity, such as in severe combined immunodeficiency (SCID) mice, mice lacking in granulocyte monocyte-colony stimulating factor (GM-CSF-/-), and NOD.CB17-Prkdc^scid^/NCrCrl mice with compromised B and T lymphocytes and natural killer cells defectively working. These conditions may result in a severe progression of *M. abscessus* infection histopathologically resembling human NTM lung disease. Conversely, single-gene deletion for NOS, ROS, TNF, IFNγ, and MyD88 alone can be compensated by the immune system [175,177,178,179]. The route of infection is suggested to greatly influence the host-immune response. For instance, aerosolized *M. abscessus* resulted in a progressive infection in IFNγ knockout (GKO) mice [176]. Otherwise, mice infected intravenously were able to control the infection [175]. Overall, experimental infection is more frequently induced by administration of 1 × 10^5^–10^9^ CFUs through inhalation of NTM-infected aerosol, the most likely route of natural pulmonary infection. Nevertheless, subcutaneous inoculation is the route of choice for inducing cutaneous disease [180], while intraperitoneal inoculation is preferred for MAP [181].

Contrary to *M. abscessus*, immunocompetent mouse strains serve as excellent models for virulent SGM, including MAC. However, the beige mutation in C57BL/6 mice led to a reduced influx of neutrophils to the site of infection, mitigating the disease development. The nude and BALB/c proved to be more appropriate model systems than C57BL/6 and beige mice to evaluate the host susceptibility to *M. avium* aerosolization and the effect of antimicrobial therapies. Indeed, while nude mice were highly susceptible to aerosol infection, BALB/c were more suited to evaluate the impact of antibiotic treatment [182]. 

Several host-protective factors appeared to modulate MAC infection, particularly nitric oxide synthase knockout (iNOS-/-) and IFNγ/TNFα treatment [183]. However, contemporary and subsequent studies partially contradicted the findings above [184,185]. 

Following the date of publication of the reviews mentioned earlier, some authors addressed the need for an animal model for the preclinical development of therapies to treat *M. abscessus* infection. As a result, a new preclinical model of *M. abscessus* was developed using aerosolized immunocompetent mice treated with corticosteroids [186]. The transient pharmacologically-induced immune suppression promoted the increase of the bacterial lung burden after implantation of *M. abscessus* and allowed to recapitulate salient features of pulmonary *M. abscessus*, including organized lesions with the influx of macrophages, neutrophils, and lymphocytes. Additionally, the need for an animal model that demonstrates a similar pulmonary disease as humans infected with MAC incited to exploit the “super-susceptibility to tuberculosis” murine model C3HeB/FeJ. This murine strain developed a progressive infection characterized by small foci of necrosis in the lungs after a lower bacterial inoculum than that typically used [187].

It is also worth mentioning the attempts of some authors to investigate nonhuman primates as a model of NTM pulmonary disease [188,189]. Although cost consideration makes these studies hardly practicable for large samples, immunecompetent monkeys could provide a unique opportunity for immunity research and drug development against age and gender-related NTM disease.

Despite the pitfalls related to the use of the above-described models, the in vivo approach has many practical and valuable applications in the field of pharmaceutical technology. In particular, the efficacy of different drug delivery routes can be investigated. In this respect, several researchers have conducted studies on inhaled antibiotics, which can be delivered to mice by commercially available micro sprayers or adapted inhalation apparatus [26,42,190].

## 8. In Vivo Preclinical Models in Vaccine Research

To date, there are no recommended vaccinal protocols neither established preclinical models to study the efficacy of potential vaccines against the wide variety of NTM. Conversely, animal models for anti-TB vaccine research are well documented, including vertebrates and invertebrates [191]. As suggested previously [192], strategies currently being considered for immunization against *M. tuberculosis* could be exploited against NTM as well. In particular, the use of Bacillus Calmette–Guérin (BCG) vaccine has been controversially taken into consideration. Indeed, BCG administered before NTM exposure showed protection against *M*. *avium* and *M*. *kansasii* in AB6 (A/Tru x C57BU6) hybrid female mice [193], but the efficacy of BCG vaccination can be highly variable, depending on earlier mycobacterial exposure [194]. Moreover, BCG was ineffective against *M*. *intracellulare* and *M*. *simiae.*

Different mouse models and ex vivo approaches have been proposed for NTM vaccine research. For instance, the capability of BCG-stimulated T cells to inhibit the intracellular replication of *M. avium* and *M. abscessus* was investigated in co-cultures with infected human autologous macrophages [195]. In the same study, BCG-induced *M. avium* cross-reactive immunity was assessed in splenic cells from immunocompetent C57BL/6 mice after challenging in vitro with BCG or *M. avium* antigens. A similar strategy was applied by others that collected blood and organs from BALB/c and BALB/c nude for CFU enumeration, IFN-γ Enzyme-Linked ImmunoSpot assay, cytotoxic T lymphocyte assay, cytokine ELISA, and antibody determination, to demonstrate the utility of *Mycobacterium paragordonaeas* as a live attenuated vaccine against *M. abscessus* and *M. tuberculosis* [196]. Genetic manipulation of murine lines made it possible to specifically address the need for effective immunization strategies against *M. abscessus* in patients with cystic fibrosis [197]. The authors used homozygote ΔF508 FVB female mice bearing the cystic fibrosis transmembrane conductance regulator (CFTR) _F508 mutation.

Novel vaccine candidates have been proposed for *M. kansasii* as well. Notably, a new vaccine consisting of B cells transduced with a vaccinia virus expressing ESAT6 and a ligand of invariant NKT cells showed inhibition of the pulmonary disease caused by *M. kansasii* infection in C57BL/6 mice [198].

Several studies in the broader literature have focused attention on vaccine development against *M. ulcerans*, recently reviewed by Bolz and Ruf [173], and will thus only be briefly mentioned here. Addressing vaccine research for *M. ulcerans* prophylaxis can be challenging because the vaccination before infection of mice prolongs the overall time of the animal experiments, which often lasts for several months. Additionally, the efficacy of the vaccination may vary with both types of pathogens and mice strains. To the best of our knowledge, BALB/c and C57BL/6 have been more frequently used [180,199].

Finally, animal models of paratuberculosis deserve mention. Several types of experimental models have been used to study Johne’s disease. However, the majority of published vaccine research exploited ruminant models, which are naturally susceptible animals to MAP infection. In the preclinical phase, the suitability of different species of laboratory animals has been tested for studying Johne’s disease and vaccinal strategies [174]. Notably, C57BL/6 and BALB/c were suggested to be useful and convenient models for studies of immunoregulation of paratuberculosis [200,201], and, thus, they have been widely used [181,202,203,204]. Conversely, a commercially available whole-cell vaccine showed inferior protection in a murine model than in rabbits. Therefore, the rabbit model could closer emulate the response to the vaccination previously observed in ruminants [205].

## 9. Concluding Comments

Most of the current strategies of NTM drug discovery follow trends previously paved by TB research. One needs only to think about HFS of intracellular pulmonary infection. While we can benefit from the knowledge and models developed for TB, the remarkable interspecies differences among NTM are responsible for the inconsistent agreement concerning the indications for susceptibility testing. Moreover, further work needs to be done to establish whether unfavorable micro-environmental conditions inside the mycobacterial lesions may trigger the growth of subpopulations of NTM and render them phenotypically resistant to drugs. Insufficient funding and long time required for mycobacterial experiments oppose the advance of knowledge. However, increasing research efforts is crucial to provide not yet developed reproducible strategies aimed at improving drug selection. By addressing these challenges, models of host-pathogen interaction can be implemented to fill the gaps in the workflow of NTM drug discovery and notably to grow neglected opportunities for vaccine research and development.

## Figures and Tables

**Table 1:** Common nontuberculous mycobacterial pathogens and their host-associated tissue tropism.

Species	Reported Tropism	Host
*Mycobacterium avium* complex *Mycobacterium kansasii* *Mycobacterium xenopi* *Mycobacterium malmoense* *Mycobacterium smegmatis*	Lungs, lymph nodes, skin and soft tissues, bone and joint, nervous system, disseminated disease	Human Occasionally, varied wild and domestic animals
*Mycobacterium avium* subsp. *paratuberculosis*	Gastroenteritis	Wild and domestic ruminants
*Mycobacterium ulcerans*	Skin and soft tissues	Human Occasionally, varied wild and domestic animals
*Mycobacterium marinum*	Skin and soft tissues	Fish Human Rarely, other wild and domestic animals
*Mycobacteroides abscessus* complex	Lungs, skin and soft tissues, bone and joint, lymph nodes, nervous system, ocular infections, disseminated disease	Human Occasionally, varied wild and domestic animals
*Mycolicibacterium fortuitum*	Skin and soft tissues, bone and joint, lungs, lymph nodes, nervous system, disseminated disease	Human Occasionally, varied wild and domestic animals

**Table 2:** Methods for high throughput extracellular testing of nontuberculous mycobacteria.

Assay	Culture Condition	Bacteria	Length (Days)	Read-Out	Reference
Microdilution	37 °C; enriched CAMHB	SGM	7–14	Visible growth	[13]
Microdilution	30 ± 2 °C; CAMHB	RGM	2–5	Visible growth	[13]
MGIT 960	37 °C; enriched 7H9B	SGM	Within 15	Fluorometric	[17]
Alamar blue	37 °C; enriched 7H9B	SGM	8	Colorimetric	[20]
Alamar blue	Nutrient starvation	RGM	7	Fluorometric	[24]
Resazurin	37 °C; 7H9B	SGM	9	Colorimetric	[22]
Tetrazolium	37 °C; enriched 7H9B	SGM	3–9	Colorimetric	[21]

Abbreviations: SGM, slow-growing mycobacteria; RGM, rapid growing mycobacteria; CAMHB, cation adjusted Mueller Hinton broth; 7H9B; 7H9 broth.

**Table 3:** Common macrophagic and epithelial infection models for nontuberculous mycobacteria drug testing.

Cell Type	PMA	Bacteria	MOI	Reference
Human THP-1	60–500 ng/mL for 24–48 h	MAC	10 for 1 h	[26,27]
		*Mycobacteroides abscessus*	1 or 10 for 1–3 h	[26,27,29]
Human U937	1 µg/mL for 24 h	MAC	5 for 4 h	[30]
Human A549	NA	MAC	10 for 2 h	[38]
	NA	MAC	10 or 20 for 2–3 h	[36,37]
Murine J774	NA	*Mycobacterium smegmatis*	1 for 3 h	[43]
	NA	MAC	5 for 4 h or 20 for 3 h	[40,42,74]
	NA	*Mycobacteroides abscessus*	5 for 4 h	[40]
Murine RAW264.7	NA	*Mycobacteroides abscessus*	10 for 2 h	[41]
BMDMs	NA	MAC	1 for 4–5 h	[50]
	NA	*Mycobacteroides abscessus*	3 for 4 h	[49]

Abbreviations: BMDMs, murine primary bone marrow-derived macrophages; MAC, *Mycobacterium avium complex*; MOI, multiplicity of infection; PMA, phorbol 12-myristate 13-acetate; NA, not applicable.

**Table 4:** Applications of the hollow-fiber system for the optimization of therapeutic regimens against nontuberculous mycobacteria.

Infection Model	Drugs Tested in the Hollow-Fiber Model	Reference
*Mycobacterium avium* subsp. *hominissuis* (ATCC 700898)	Azithromycin plus ethambutol and rifabutin	[101]
	CARTM ^1^ regimen	[101]
	Ceftaroline Ceftazidime plus avibactam	[102]
	Ethambutol	[92]
	Ethambutol plus azithromycin	[96,103]
	Linezolid	[104]
	Minocycline	[105]
	Moxifloxacin	[106]
	Tedizolid	[107]
	Thioridazine	[108]
	Thioridazine plus azithromycin	[96]
	Thioridazine plus moxifloxacin	[96]
*Mycobacteroides abscessus* (ATCC 19977)	Amikacin	[94]
	Amikacin plus cefoxitin and clarithromycin	[109]
	Moxifloxacin	[110]
	Tigecycline	[111]
*Mycobacterium kansasii* (ATCC 12478)	Clofazimine	[112]
	Clofazimine plus an efflux pump blocker	[112]
	Isoniazid plus rifampin and ethambutol	[95,112]
	Moxifloxacin	[95]
	Moxifloxacin plus an efflux pump blocker	[95]

^1^ ceftazidime/avibactam, rifabutin, tedizolid and moxifloxacin regimen.

## Supplementary Materials

The following are available online at https://www.mdpi.com/2076-0817/9/8/641/s1, Figure S1: PRISMA 2009 Flow Diagram showing the results of the database search.
