# Supplementary figures and images for: Preclinical Models of Nontuberculous Mycobacteria Infection for Early Drug Discovery and Vaccine Research
Source: Pathogens. 2020 Aug 6;9(8):641. doi: 10.3390/pathogens9080641 (PMC7459799; doi:10.3390/pathogens9080641)

**Figure S1.** PRISMA 2009 Flow Diagram showing the results of the database search.

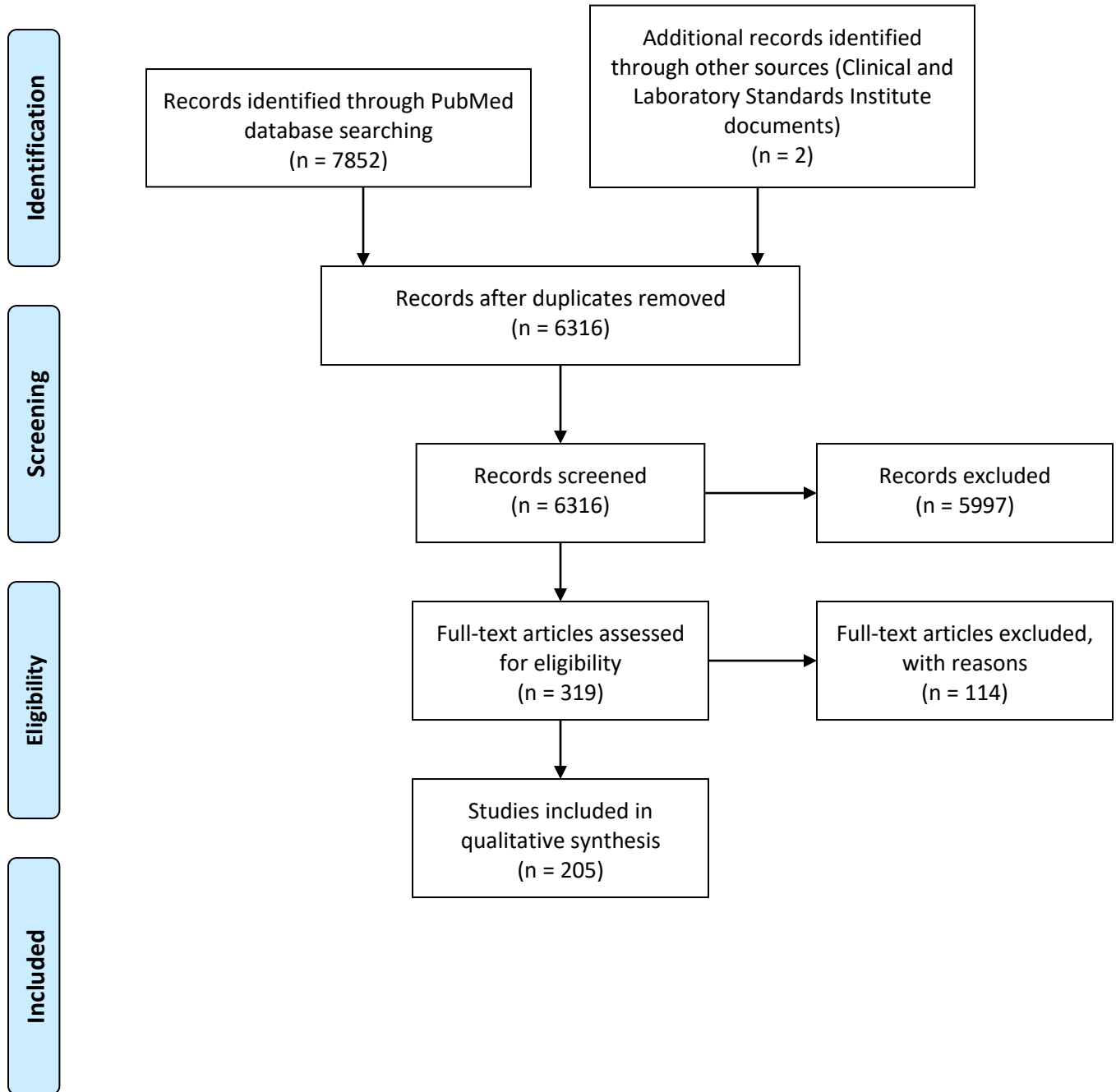

Supplement: Supplementary file 1 [file pathogens-09-00641-s001.pdf]
